# Auditory figure-ground analysis in rostral belt and parabelt of the macaque monkey

**DOI:** 10.1038/s41598-018-36903-1

**Published:** 2018-12-18

**Authors:** Felix Schneider, Pradeep Dheerendra, Fabien Balezeau, Michael Ortiz-Rios, Yukiko Kikuchi, Christopher I. Petkov, Alexander Thiele, Timothy D. Griffiths

**Affiliations:** Institute of Neuroscience, Newcastle University, Framlington Place, Newcastle upon Tyne, NE2 4HH United Kingdom

## Abstract

Segregating the key features of the natural world within crowded visual or sound scenes is a critical aspect of everyday perception. The neurobiological bases for auditory figure-ground segregation are poorly understood. We demonstrate that macaques perceive an acoustic figure-ground stimulus with comparable performance to humans using a neural system that involves high-level auditory cortex, localised to the rostral belt and parabelt.

## Introduction

Figure-ground analysis is critical to making sense of the natural world. This is a particularly challenging problem in the auditory system where different sound objects emanating from the same spatial location have to be dynamically decoded using spectro-temporal features that are difficult to segregate from noisy backgrounds^1,2^. We assessed the perception and neural representation of auditory figure-ground stimuli in the macaque. Macaques have similar audiograms^3^, detection of tones in quiet^4^, detection of tones in noise^5^ and similar pitch perception^6^ to humans. Macaques also show homologous organisation of the auditory cortex that allows comparison with that in humans^7,8^. The aims of the study were twofold: to establish whether macaques can carry out acoustic figure-ground segregation like humans and to define the areal organisation for analysis in auditory cortex.

**Figure 1:**
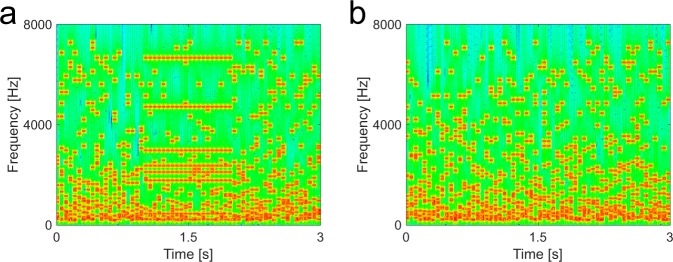
Spectrogram of stochastic figure-ground (SFG) stimulus used for behavioural experiments. (**a**) Stimulus contains a 1 s figure with a coherence level (number of channels with repeated elements) of 8 components and a figure onset set to 1 s. Each chord comprised the same number of elements (n = 15). (**b**) Control stimulus without figure.

We used a stimulus in which a figure emerges from a noisy background^9^ (Fig. 1). The paradigm captures a high-level acoustic process that requires grouping over frequency and time in complex sounds devoid of species-specific meaning, such as speech. The stochastic figure-ground (SFG) stimuli consist of multiple randomly generated frequency elements where a foreground object, arising from the grouping of different frequency elements over time, can only occur if coherently repeated elements are present in a number of frequency channels. A series of human behavioural and modelling experiments is consistent with a grouping mechanism based on temporal coherence between the frequencies comprising the figure^9^. Human imaging experiments using fMRI^10^ and MEG^11^ demonstrate activity in non-primary auditory cortex corresponding to figures that are perceived, but whether the same would hold behaviourally and neurobiologically in an animal model is unknown.

## Results

**Figure 2:**
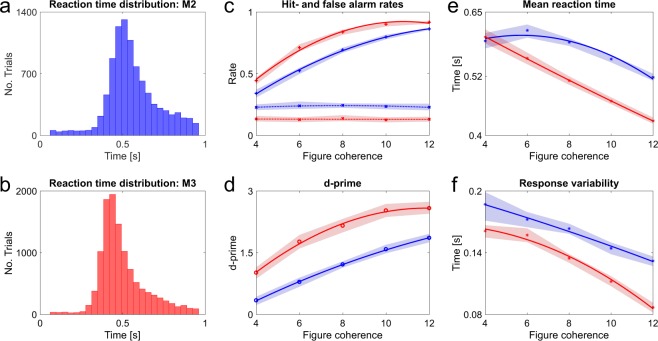
Summary of behavioural performance for active figure detection task. All data colour-coded: Blue –M2, Red – M3. (**a**) Reaction time histogram across all coherence conditions for M2 (**b**) and M3. RT data are corrected for sound output latency. (**c**–**f**) Mean values across all sessions shown for each coherence condition and subject. Shaded areas represent 95% confidence intervals. Solid and dashed lines show fitted data for each monkey, respectively. (**c**) Hit rates (solid line) and false alarm rates (dashed lines). (**d**) D-prime values. (**e**) Mean reaction times. (**f**) Response variability. Results shown in **d**, **e** and **f** were tested via repeated measures ANOVA and were significant beyond p < 0.001. See Supplementary Information.

Behavioural experiments tested if macaques can segregate such complex auditory figures. Two monkeys were trained to perform an active figure detection task. Proficiency on the task is indicated by the mean hit rates to the most salient condition with figures comprising 12 coherent frequency elements (M2: 0.86, M3: 0.92). The reaction time (RT) distributions show a clear peak for both subjects (Fig. 2a,b, M2: Peak bin: 0.49 s–0.53 s, Mean RT: 0.56 s; M3: Peak bin: 0.42 s–0.46 s, Mean RT: 0.50 s), indicating competent detection of auditory figures. Hit rates (Fig. 2c) increased as a function of figure coherence. False alarm rates were constant across coherence conditions, suggesting that monkeys could competently withhold responses to stimuli without a figure. D-prime values mirror the trend of hit rates with increasing values for more salient figures (Fig. 2d). The effect of figure coherence is significant (Repeated measures ANOVA: F(4, 200) = 266.67, p = 5.84e^−79^), indicating that the number of coherent elements has an impact on the detection performance throughout sessions. Furthermore, we found decreasing reaction times and response variability with increasing saliency of the figures (Fig. 2e,f, Mean RT: Repeated measures ANOVA, Lower bound correction applied: F(1, 50) = 253.89, p = 3.12e^−21^; Response variability: Repeated measures ANOVA, Lower bound correction applied: F(1, 50) = 142.88, p = 2.85e^−16^). The RT distributions also indicate that the detection threshold of both macaques seems to be around a coherence level of four elements, albeit coherence levels lower than four were not tested. Humans can detect these figures given an adequate figure duration^9^. Overall, the behavioural performance indicates that macaques can perceive auditory figures in noisy acoustic scenes and that behavioural performance increases with signal to noise ratio, as is the case for human listeners^9^.

**Figure 3:**
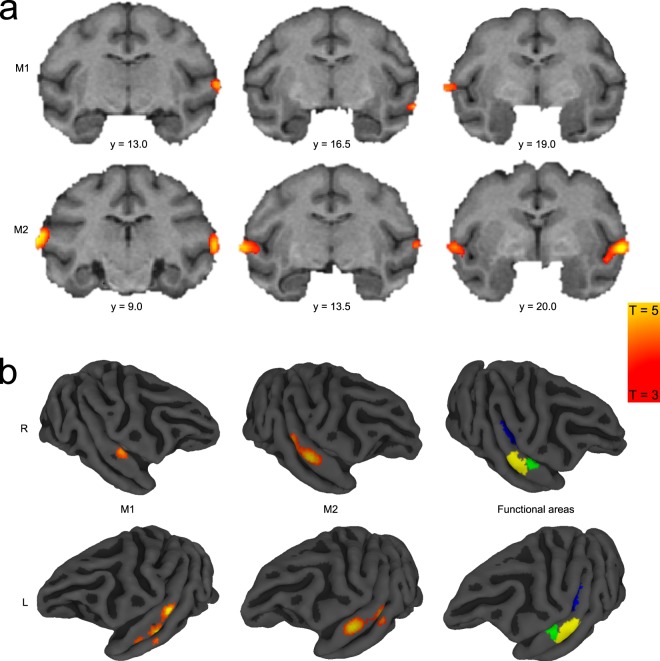
Figure vs Control contrast overlaid on standard brain. (**a**) Series of coronal MR images from posterior (left) to anterior (right) with Figure vs Control contrast overlay (3 < T < 5) for subject M1 (above) and M2 (below). Position of slices relative to interaural line in [mm] is indicated below slices. (**b**) Figure vs Control contrast overlaid on right (above) and left (below) brain surface of M1 (left) and M2 (middle). Colour-coded probabilistic maps of functional areas overlaid on standard brain (right). Functional areas: A1 - Primary auditory cortex (blue), RPB - Rostral parabelt (yellow), RTL - Lateral rostrotemporal area (green).

**Figure 4:**
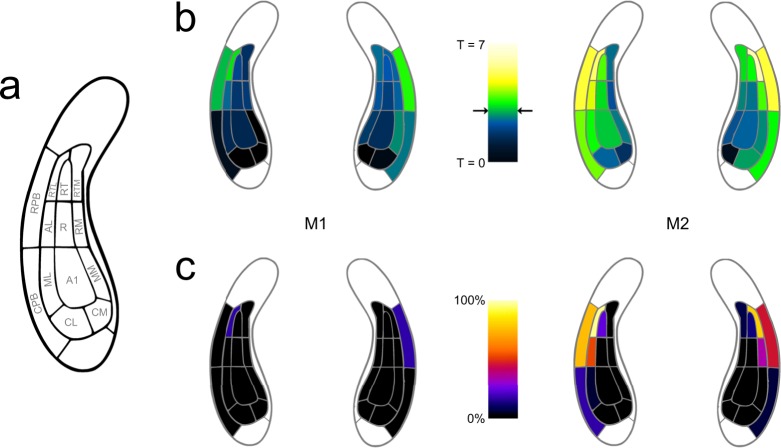
Involvement of auditory areas in figure-ground processing. (**a**) Map of macaque auditory cortex. (**b**) Maximum T-values for Figure vs Control contrast overlaid on auditory fields for M1 (left) and M2 (right). Data based on probabilistic maps. Significance level of T = 3 is indicated by black arrows. (**c**) Fraction of significant voxels per auditory field.

We acquired fMRI data from two naïve monkeys during passive exposure to the SFG stimuli. Functional imaging data were recorded before the same animals were trained in the active figure detection task. A contrast for Figure vs Control (p < 0.001, uncorrected, Fig. 3 + Table 1) revealed significant results at the convexity of the superior temporal gyrus and at the rostral parts of the superior temporal plane, demonstrating bilateral involvement of higher-level auditory regions rostro-laterally to the auditory core. These results are in line with previous human studies, showing cortical responses in non-primary auditory cortex^10,11^. In order to assign a functional area to the peak BOLD response, we illustrate the Figure vs. Control contrast along with probabilistic functional maps of auditory cortical fields, which were derived from tonotopic gradients of six macaques. This comparison reveals that the main activation during a perceived figure is located in the rostral parabelt (RPB) and the rostro-lateral belt (RTL) for both monkeys (Fig. 4). The significant clusters also extend to the rostral superior temporal gyrus (STGr), the rostral core (RT), the anterolateral belt (AL) and the caudal parabelt (CPB). In addition, we find that T-values ramp up towards the rostro-lateral parts of the auditory field. Thus, we conclude that figure-ground processing happens in rostral parts of the auditory ventral stream.

**Table 1:** Coordinates of maximum Figure vs Control contrast in M1 and M2 for each hemisphere. Data are displayed relative to interaural line.

Subject	Hemisphere	X [mm]	Y [mm]	Z [mm]
M1	L	29	13	14
	R	−29	18.5	12
M2	L	29	21.5	10.5
	R	−27	17	12

## Discussion

This work establishes the ability of macaques to carry out dynamic figure-ground segregation with remarkably similar psychometric functions to humans^9^. Neural correlates of auditory scene analysis have previously been found in primary auditory cortex for two-tone paradigms^12,13^, however, we demonstrate a system involving circumscribed parts of the rostro-lateral belt and parabelt cortex, at a high level in the cortical hierarchy in macaques^14–16^ for complex figure-ground segregation. In line with our results, previous evidence suggests that the most anterior regions of the ventral processing stream represent a complete acoustic signature of auditory objects^17^.

Although we demonstrate that macaques are able to carry out the behavioural task, there is a difference in the sensitivity to figures between humans and macaques. Whilst humans are reliably able to segregate figures with a coherence level of two elements^9^, performance for our two subjects was worse for figures with a higher coherence level and longer duration. However, the overall trend of detection performance as a function of coherence was the same.

We have argued that the detection of the SFG stimulus requires a mechanism that can integrate across different frequency bands in order to detect temporal coherence between these^9^. A possible mechanism of figure-ground analysis is based on single neurons in high-level cortex with inputs from combinations of units in primary cortex with narrowband or multi-peaked tuning: neuronal responses to sounds with harmonically related components are described in primate core^18^ and belt areas^19^. However, a neuronal mechanism for the present results requires neurons to respond to multiple frequencies that do not have a simple mathematical relationship to each other. One imaging study suggests harmonic and non-harmonic multipeak tuning in large parts of the ventral auditory stream^20^. fMRI BOLD, however, does not allow disambiguation of such a neuronal mechanism from a population code. The necessary broadband tuning for such units is well described in the belt cortex^19,21^. Broadband responses in the parabelt are likely given they occur at a high level in the auditory hierarchy^14–16^ but receptive fields of parabelt neurons have not been extensively characterised^22^. From first principles such neurons might be expected at a high level in the auditory hierarchy: we predict the existence of such units in the rostro-lateral belt and parabelt. In a later stage, the grouping of repeated elements and detection of the figure could cause top-down modulation in upstream brain areas like A1 in the form of neural entrainment^23^.

Previous studies have found an involvement of the intraparietal sulcus (IPS) in stream segregation^24^ and figure-ground processing^10,11^. Contrary to these studies, we were not able to show a BOLD response modulation in the IPS, which could be due to the cranial implants of the animals that can lead to signal dropouts. Alternatively, a species differences in figure-ground processing cannot be ruled out.

In summary, our data suggest that a fundamental form of figure-ground analysis is perceived both by macaques and humans and relies on non-primary auditory cortex in both species. Our approach allows us to investigate grouping over frequency-time space using stimuli that are not species-specific, but which require grouping mechanisms that are relevant to the extraction of species-relevant sounds from noise. This work predicts specific neuronal responses to figure-ground analysis in rostro-lateral auditory areas that can now be investigated systematically in the macaque in a way that is not possible in humans.

## Methods

All procedures performed in this study were approved by the UK Home Office (Project License: 70/7976) and by the Animal Welfare and Ethical Review Body at Newcastle University. All experiments comply with the UK Animals Scientific Procedures Act (1986) on the care and use of animals in research, with the European Communities Council Directive on the protection of animals used in research (2010/63/EC) and with the US National Institute of Health Guidelines. We support the principles of the consortium on Animal Research Reporting of *In Vivo* Experiments (ARRIVE).

### Animals

Three adult macaques (*Macaca mulatta*, one female) were used in this study. Both males contributed to the imaging data set. One male and one female monkey were included in the behavioural tests (see Table 2). M1 was not available for figure detection training. M3 does not have a cranial implant which is a necessary prerequisite for awake fMRI scans. Animals were kept under fluid controlled conditions. Fluid control was within ranges which do not negatively affect animal’s physiological or psychological welfare^25^.

**Table 2:** Summary of subjects participating in imaging and behavioural experiments.

Animal ID	Gender	Age [years]	Weight [kg]	Imaging	Behaviour
M1	Male	11	9	Y	N
M2	Male	11	11	Y	Y
M3	Female	5	6	N	Y

### Stimuli

Stochastic Figure-Ground (SFG) stimuli were created at a sampling rate of 44.1 kHz with MATLAB (The Mathworks Inc., Natick, MA). Signals consisted of a sequence of 50 ms long chords, defined as a sum of multiple pure tone elements that were not harmonically related. The onset and offset of each tone was shaped by a 10 ms raised-cosine ramp. Some stimuli included a sequence of repeated elements within several frequency channels (‘figure’). The remaining signals comprised randomly shuffled elements only (‘control’).

For functional imaging, stimuli consisted of 120 chords (6 s in duration) in total. For each of these chords a random number of 5 to 15 tonal ground elements was drawn from a pool containing 129 evenly spaced frequencies (1/24 octave between successive frequencies) on a logarithmic scale between 179 Hz and 7246 Hz. The number of bands that contribute to the figure (‘coherence’) was set to a constant value (n = 10). SFG stimuli used for imaging had extra coherent or shuffled elements added on top of the ground signal after two seconds for the following 40 chords (2 s in duration). Stimulus parameter are consistent with previous studies^9–11^.

For behavioural testing, stimuli contained 60 chords (3 s in duration) and had a fixed number of elements per chord (n = 15). In contrast to the imaging stimuli, extra elements were not added on top but incorporated into the existing stream of chords to remove any sound level cues. The coherence level of the figure was varied between 4, 6, 8, 10 and 12 elements. Figure onset times were randomised between 0.3 and 2 seconds. For both experiments, figure and control stimuli were presented in a randomised order.

### Behavioural training

All subjects were naïve to the behavioural detection task. By means of positive reinforcement, we established a bar release – reward relationship. Since subjects needed to be trained in a detection task, a fixed target stimulus was paired via operant conditioning. This target was a plain figure (duration: 1000 ms, coherence: 10) without any distractor elements. After monkeys responded proficiently to the sound, we introduced the SFG background tones. The signal to noise ratio was gradually decreased by increasing the sound level of the ground signal. Subsequent to this introductory phase, the ground sound intensity was set to a fixed level (65 dB) whereas the figure sound level was incremented to give subjects an extra cue to the target. These sound level increments were then gradually decreased until subjects could detect the figures without any intensity cues. As a last step, figure coherence was manipulated in order to assess the animal’s performance. The entire training period took around 8 months of daily training.

### Experimental design: Behavioural paradigm

To make inferences about the streaming ability of macaques in crowded acoustic scenes, we designed a figure detection task as a Go/No-Go paradigm. For behavioural testing, macaques sat in a primate chair (Crist Instruments) and initiated trials by touching a touch bar, placed in front of them. Two free-field speakers (Yamaha Monitor Speaker MS101 II), located at approximately 45 degree to the left and right of the animal (distance: ~65 cm from ear), delivered the stimuli at ~65 dB SPL via an Edirol UA-4FX external USB-Soundcard. The experiment was controlled with a custom made MATLAB (2015b) script, including PsychToolbox 3.0 functions through a LabJack U3-HV interface.

Before each session, a new set of stimuli was created (n = 1000). For each trial, a stimulus file was randomly drawn from this pool of stimuli. If the monkey responded correctly during the figure presentation period (‘Hit’), a fluid reward was administered through a gravity based reward system. The amount of reward was dependent on the reaction time of the respective trial. Faster responses led to higher reward volumes. Inter-trial intervals (ITI) were set to 1 s. In case the monkeys missed to respond to a target, no reward was administered but a 3 s penalty time-out was imposed in addition to the ITI. Stimuli were terminated as soon as the subjects responded or after the target sound ended. Trials with stimuli containing a figure comprised 60% of all trials. The remaining 40% were catch trials (control condition) in which only the ground stimulus was presented. In these catch trials, subjects needed to hold the touch bar for the entire length of the stimulus (3 s). In case of a correct rejection of the trial (bar not released), a fixed reward was given. The amount of juice earned on those trials was greater than during detection trials, since monkeys had to hold the bar up to two seconds longer. Similar to the miss of a figure, false alarms resulted in no reward but a 3 s penalty time-out in addition to the ITI. Each behavioural sessions lasted around two hours (average number of trials per session: M2 = 1000, M3 = 873). Data were acquired, saved and analysed using MATLAB.

### Experimental design: Imaging paradigm

For functional imaging scans, macaques were transferred into a custom-made, MRI-compatible scanner chair. During the session, awake animals were head restrained by means of an implanted head post. The details of the surgical procedures are described in Thiele *et al*. (2006)^26^. Single-shot echo-planar images were acquired with an actively shielded, vertical 4.7T MRI scanner (BrukerBiospec 47/60 VAS) equipped with a Bruker BGA-38S gradient system with an inner-bore diameter of 38 cm (BrukerBioSpin GmbH, Ettlingen, Germany). One volume transmit coil and two 4 channel receiver coils were used. A sparse imaging paradigm was applied to avoid the interfering effect of the high intensity noise generated by the MRI scanner. Shimming was performed with the MAPSHIM algorithm^27^ which measures B0 field inhomogeneity to apply first and second order corrections to it. The applied sequence was a GE-EPI with 2x GRAPPA acceleration with the following parameters: TR = 10 s, TA = 2011ms, TE = 21 ms, flip angle (FA) of 90°, receiver spectral bandwidth of 200 kHz, field of view (FOV) of 9.6 × 9.6 cm^2^, with an acquisition matrix of 96 × 96, an in plane resolution and slice thickness of 1.2 mm and 32 slices. The TR duration was sufficient to avoid recording the BOLD response to the gradient noise of the previous scan. Per scan 360 volumes were acquired (of which 90 volumes baseline/silence).

In total, 135 stimuli per condition (control i.e. ground only or figure) were created and presented in pseudo-randomized manner. The same stimuli were used for all scans and all subjects. Sounds were presented using Cortex software (Salk institute) at an RMS sound pressure level (SPL) of 75 dB via custom adapted electrostatic headphones based on a Nordic NeuroLab system (NordicNeuroLab, Bergen, Norway). These headphones feature a flat frequency response up to 16 kHz and are free from harmonic-distortion at the applied SPL. SPL was verified using an MR-compatible condenser microphone B&K Type 4189 (Bruel&Kjaer, Naerum, Denmark) connected by an extension cable to the sound level meter Type 2260 (same company). A structural scan was acquired at the end of each functional scanning session. Anatomical MR images are T1-weighted (T1w) images, consisting of a 2D magnetization-prepared rapid gradient-echo (MPRAGE) sequence with a 180° preparation pulse, TR = 2000 ms, TE = 3.74 ms, TI = 750 ms, 30° flip angle, receiver bandwidth = 50 KHz, an in-plane resolution of 0.67 × 0.67 mm^2^ with a slice thickness of 0.6 mm. Structural scans covered the same field of view as the functional scans.

### Statistical analysis: Behaviour

For data analysis, signal detection theory was applied. In total, data from 52 behavioural sessions were included in this analysis (M2: 23, M3: 29). Performance was evaluated based on hit and false alarm rates, which are the basis for d′ calculation, a measure of discriminability between responses to different stimuli. Computation of d′ values was done by using the formula below:

$$d^{\prime} =Z(Hit\,rate)-Z(False\,alarm\,rate)$$

where Z is the z-transform of hit/false alarm rate respectively, which is defined as the inverse of the cumulative Gaussian distribution (MATLAB: norminv). Since d’ values take hit rates as well as false alarm rates into account, they provide a measure of all possible responses to both detection- and catch trials. Mean d′ values across all sessions for each coherence condition were the basis for the assessment of the behavioural performance. Trials with responses below 0.4 s after stimulus onset were excluded from the analysis (M2: 1.67%, M3: 1.38%). Reaction times were corrected for sound output latency of the operating system. 95% confidence intervals were calculated via bootstrapping (MATLAB: bootci, 5000 repetitions). Data were fitted with second order polynomial function. For statistical testing, data of both subjects were pooled as we were interested in the overall trend of the responses. Effects of coherence were tested across sessions with a repeated measures ANOVA for d-prime values, mean reaction times and responses variability, respectively. Normal distribution was evaluated with a one-sample Kolmogorov-Smirnov test. A Mauchly sphericity test assessed if the assumption of sphericity was violated. If that was the case, a conservative lower bound correction was applied to the degrees of freedoms and p-values of the repeated measures ANOVA.

### Statistical analysis: Imaging

MR images were first converted from the scanner’s native file format into a common MINC file format using the Perl script pvconv.pl (http://pvconv.sourceforge.net/). From MINC format, it was converted to NIfTI file format using MINC tools. Imaging data were then analysed with SPM12 (http://www.fil.ion.ucl.ac.uk/spm/software/spm12/-Wellcome Trust Centre for Neuroimaging).

In the pre-processing steps, the volumes within a session are realigned and resliced to incorporate the rigid body motion compensation. Next, image volumes from multiple sessions were combined by realigning all volumes to the first volume of the first session. Then, this data was spatially smoothened using a Gaussian kernel with full-width-at-half-maximum (FWHM) of 3 mm. A standard SPM regression model was used to partition components of the BOLD response at each voxel. The two conditions, figure and control, were modelled as effects of interest and convolved with a hemodynamic boxcar response function. Next, the time series was high pass filtered with a cut-off of 1/120 Hz to remove low-frequency variations in the BOLD signal. Finally, this data was adjusted for global signal fluctuations also known as global scaling to account for differences in system responses across multiple sessions. A general linear model (GLM) analysis^28^ of the combined sessions included the motion parameters, the voxel-wise response estimates and the regression coefficients. The t-values for two contrasts (Figure vs Control, Sound vs Silence) were calculated. We performed single subject inference in these two subjects. Data were thresholded at p < 0.001 (uncorrected for multiple comparisons across the brain). Results from monkey M2 survived p < 0.05 (family wise error corrected across the brain) and it showed a pattern similar to that presented here. Data were coregistered and displayed in standard space (D99)^29^.

The total number of scans for the two monkeys was as follows (M1: 12, M2: 10). Sessions with obvious large imaging artefacts, high signal differences between hemispheres and/or insufficient baseline activity in the sound vs silence contrast were not included in the analyses (M1: 6, M2: 4 sessions).

### Probabilistic maps

The applied probabilistic maps are an estimate of functional areas of the auditory field in standard space (D99)^29^ based on the tonotopic gradients of six macaques (not included in this study), with the probabilistic map threshold set at 0.5, equivalent to at least 3 animals overlapping in the location of the auditory cortical fields. Isofrequency lines from mirror reversals between core (A1/R) and belt areas (ML/AL) were extended laterally to approximate the border between rostral and caudal parabelt. For each functional area, all voxels have an assigned value, representing the probability that a given voxel fell within this field. By thresholding these maps to 0.5, we made sure that each voxel is in at least 50% of the scanned population within the boundaries of the respective functional field.

Data and code available on request from the corresponding authors.

## Supplementary information

Supplementary Information

## References

[1] Bregman, A. S. *Auditory Scene Analysis: The Perceptual Organization of Sound*. *MIT Press* (MIT Press, 1990).

[2] Shamma SA, Elhilali M, Micheyl C (2011). Temporal coherence and attention in auditory scene analysis. Trends Neurosci..

[3] Jackson LL, Heffner RS, Heffner HE (1999). Free-field audiogram of the Japanese macaque (Macaca fuscata). J. Acoust. Soc. Am..

[4] Heffner HE, Heffner RS (1986). Hearing loss in Japanese macaques following bilateral auditory cortex lesions. J. Neurophysiol..

[5] Dylla M, Hrnicek A, Rice C, Ramachandran R (2013). Detection of Tones and Their Modification by Noise in Nonhuman Primates. J. Assoc. Res. Otolaryngol..

[6] Joly, O. *et al*. A perceptual pitch boundary in a non-human primate. *Front. Psychol*. **5** (2014). 10.3389/fpsyg.2014.00998 PMC4163976 25309477

[7] Baumann S, Petkov CI, Griffiths TD (2013). A unified framework for the organization of the primate auditory cortex. Front. Syst. Neurosci..

[8] Leaver AM, Rauschecker JP (2016). Functional Topography of Human Auditory Cortex. J. Neurosci..

[9] Teki S, Chait M, Kumar S, Shamma S, Griffiths TD (2013). Segregation of complex acoustic scenes based on temporal coherence. Elife.

[10] Teki S, Chait M, Kumar S, von Kriegstein K, Griffiths TD (2011). Brain Bases for Auditory Stimulus-Driven Figure-Ground Segregation. J. Neurosci..

[11] Teki S (2016). Neural Correlates of Auditory Figure-Ground Segregation Based on Temporal Coherence. Cereb. Cortex.

[12] Lu K (2017). Temporal coherence structure rapidly shapes neuronal interactions. Nat. Commun..

[13] Fishman YI, Kim M, Steinschneider M (2017). A Crucial Test of the Population Separation Model of Auditory Stream Segregation in Macaque Primary Auditory Cortex. J. Neurosci..

[14] Kaas JH, Hackett TA (2000). Subdivisions of auditory cortex and processing streams in primates. Proc. Natl. Acad. Sci..

[15] Hackett TA (2014). Feedforward and feedback projections of caudal belt and parabelt areas of auditory cortex: refining the hierarchical model. Front. Neurosci..

[16] Scott BH (2015). Intrinsic Connections of the Core Auditory Cortical Regions and Rostral Supratemporal Plane in the Macaque Monkey. Cereb. Cortex.

[17] Leaver AM, Rauschecker JP (2010). Cortical Representation of Natural Complex Sounds: Effects of Acoustic Features and Auditory Object Category. J. Neurosci..

[18] Feng L, Wang X (2017). Harmonic template neurons in primate auditory cortex underlying complex sound processing. Proc. Natl. Acad. Sci..

[19] Kikuchi Y, Horwitz B, Mishkin M, Rauschecker JP (2014). Processing of harmonics in the lateral belt of macaque auditory cortex. Front. Neurosci..

[20] Moerel M (2013). Processing of Natural Sounds: Characterization of Multipeak Spectral Tuning in Human Auditory Cortex. J. Neurosci..

[21] Rauschecker JP, Tian B (2004). Processing of Band-Passed Noise in the Lateral Auditory Belt Cortex of the Rhesus Monkey. J. Neurophysiol..

[22] Kajikawa Y (2015). Auditory Properties in the Parabelt Regions of the Superior Temporal Gyrus in the Awake Macaque Monkey: An Initial Survey. J. Neurosci..

[23] Barczak A (2018). Top-down, contextual entrainment of neuronal oscillations in the auditory thalamocortical circuit. Proc. Natl. Acad. Sci..

[24] Cusack R (2005). The Intraparietal Sulcus and Perceptual Organization. J. Cogn. Neurosci..

[25] Gray, H. *et al*. Physiological, Behavioral, and Scientific Impact of Different Fluid Control Protocols in the Rhesus Macaque (Macaca mulatta). *eNeuro* **3** (2016). 10.1523/ENEURO.0195-16.2016 PMC5032891 27679812

[26] Thiele A, Delicato LS, Roberts MJ, Gieselmann MA (2006). A novel electrode-pipette design for simultaneous recording of extracellular spikes and iontophoretic drug application in awake behaving monkeys. J. Neurosci. Methods.

[27] Kanayamay S, Kuhara S, Satoh K (1996). *In vivo* rapid magnetic field measurement and shimming using single scan differential phase mapping. Magn. Reson. Med..

[28] Friston KJ (1994). Statistical parametric maps in functional imaging: A general linear approach. Hum. Brain Mapp..

[29] Saleem, K. S. & Logothetis, N. K. *Atlas of the Rhesus Monkey Brain in Stereotaxic Coordinates*. *Academic Press* (2012).

