# Supplementary material for: Auditory figure-ground analysis in rostral belt and parabelt of the macaque monkey
Source: Sci Rep. 2018 Dec 18;8:17948. doi: 10.1038/s41598-018-36903-1 (PMC6298974; doi:10.1038/s41598-018-36903-1)
Supplement: Supplementary file 1 — Supplementary Information [file 41598_2018_36903_MOESM1_ESM.docx]

**Auditory figure-ground analysis in rostral belt and parabelt of the macaque monkey**

**Supplementary Information**

Felix Schneider^1+*^, Pradeep Dheerendra^1+*^, Fabien Balezeau^1^, Michael Ortiz-Rios^1^, Yukiko Kikuchi^1^, Christopher I Petkov^1^, Alexander Thiele^1^, Timothy D Griffiths^1^

^1^Institute of Neuroscience, Henry Wellcome Building, Newcastle University Medical School, Framlington Place, Newcastle upon Tyne, NE2 4HH

^+^ These authors contributed equally.

* Correspondence: felix.schneider@ncl.ac.uk; pradeep.dheerendra@gmail.com;

S1 Table: Behavioural data M2

| **Coherence level** | **4** | **6** | **8** | **10** | **12** |
| --- | --- | --- | --- | --- | --- |
| **Mean hit rates** | 0.3405 | 0.5248 | 0.6935 | 0.7979 | 0.8633 |
| **Mean false alarm rates** | 0.2275 | 0.2397 | 0.2440 | 0.2347 | 0.2290 |
| **Mean d-prime** | 0.3387 | 0.7877 | 1.2121 | 1.5812 | 1.8585 |
| **Mean reaction time [s]** | 0.5914 | 0.6129 | 0.5888 | 0.5547 | 0.5178 |
| **Mean response variability [s]** | 0.1871 | 0.1724 | 0.1635 | 0.1443 | 0.1320 |

S2 Table: Behavioural data M3

| **Coherence level** | **4** | **6** | **8** | **10** | **12** |
| --- | --- | --- | --- | --- | --- |
| **Mean hit rates** | 0.4444 | 0.7118 | 0.8381 | 0.9012 | 0.9173 |
| **Mean false alarm rates** | 0.1335 | 0.1278 | 0.1375 | 0.1257 | 0.1311 |
| **Mean d-prime** | 1.0129 | 1.7647 | 2.1545 | 2.5238 | 2.5801 |
| **Mean reaction time [s]** | 0.5993 | 0.5563 | 0.5110 | 0.4696 | 0.4294 |
| **Mean response variability [s]** | 0.1611 | 0.1572 | 0.1346 | 0.1120 | 0.0869 |
